# The multiple roles of nerve biopsy in the diagnosis and prognosis of suspected immune neuropathies

**DOI:** 10.1007/s00415-024-12456-4

**Published:** 2024-05-29

**Authors:** Rafael Klimas, Anna Kordes, Sophie Huckemann, Zornitsa Gasz, Jörg Philipps, Melissa Sgodzai, Thomas Grüter, Melis Sevindik, Christiane Schneider-Gold, Ralf Gold, Kathy Keyvani, Min-Suk Yoon, Anna Lena Fisse, Kalliopi Pitarokoili, Jeremias Motte

**Affiliations:** 1 Department of Neurology, St. Josef-Hospital, Ruhr-University Bochum, Gudrunstrasse 56, 44791 Bochum, Germany; 2 Immune-Mediated Neuropathies Biobank (INHIBIT), Ruhr-University Bochum, Bochum, Germany; 3 Department of Internal Medicine, Rhein-Maas Klinikum, Würselen, Germany; 4 Department of Neurology and Neurogeriatrics, Johannes-Wesling-Klinikum Minden, Ruhr-University, Bochum, Germany; 5 Department of Neurology, Evangelic Hospital Lippstadt, Lippstadt, Germany; 6 Department of Neurology, Evangelisches Krankenhaus Hattingen, Hattingen, Germany; 7 Institute of Neuropathology, University of Duisburg-Essen, Essen, Germany

**Keywords:** Polyneuropathy, Nerve biopsy, CIDP, Histology

## Abstract

**Introduction:**

The value of a sural nerve biopsy for the diagnosis of chronic inflammatory demyelinating polyradiculoneuropathy (CIDP) is controversial. Evidence-based recommendations for its implementation are lacking. We investigated factors leading to biopsy and analyzed biopsy outcomes and consequences, assessed the predictability of biopsy outcomes through clinical parameters to avoid unnecessary biopsies, and compared results with electrophysiological and clinical severity to determine their prognostic value.

**Methods:**

190 sural nerve biopsies were analyzed in two cohorts. One consisted of 163 biopsies and the second of 72 biopsies from the prospective Immune-mediated Neuropathies Biomaterial and Data registry (INHIBIT). Both have an intersection of 45 patients. 75 data sets from patients without biopsy were used. Analysis of nerve conduction studies, treatment, overall disability sum score (ODSS), biopsy outcomes, and diagnosis was performed.

**Results:**

51% of biopsied patients received the diagnosis CIDP (77% fulfilled EFNS/PNS criteria), 21% were not CIDP typical, and 27% were unspecific. Biopsied patients responded less frequently to immunotherapies at time of biopsy than non-biopsied patients (*p* = 0.003). Immunotherapy was initiated more frequently after biopsy (*p* < 0.001) and more often with intravenous immunoglobulins (*p* < 0.0001). 76% of all biopsied patients met the electrophysiological criteria for CIDP. Sensory nerve action potential amplitudes of 0 µV still provide 73% of histological diagnostic value. Histologic signs of degeneration predicted ODSS worsening after 1 year (*p* = 0.028) but disease severity did not correlate with histological damage severity.

**Discussion:**

The main indication for nerve biopsy was the treatment of refractory cases of autoimmune neuropathies with the therapeutic consequence of treatment initiation or escalation. Sural biopsy also provided prognostic information. Even with extinguished sural SNAP, the biopsy can still have diagnostic value.

## Introduction

Chronic inflammatory demyelinating polyradiculoneuropathy (CIDP) is the most common treatable autoimmune neuropathy causing progressive or relapsing–remitting weakness and sensory impairment with a prevalence of 0.8–8.9 per 100,000 [1, 2]. The pathogenesis of CIDP is complex and incompletely understood, involving both cellular and humoral immune mechanisms leading to demyelination and axonal damage [3, 4].

The diagnosis of CIDP is challenging since the clinical presentation is heterogeneous and diagnostic markers are lacking [3]. Current diagnostic criteria for CIDP are based on clinical and electrophysiological findings, as well as the exclusion of other causes of neuropathy [5–7]. However, these criteria are not always sufficient to make a definitive diagnosis, especially in cases of advanced nerve degeneration [3, 8]. In such situations, nerve biopsy can provide valuable information that may support or confirm the diagnosis of CIDP, as well as provide insight into the pathophysiologic processes and prognostic factors [9]. Nerve biopsy can invasively reveal characteristic features of CIDP, such as demyelination, inflammation, onion bulb formation, and axonal loss [10, 11]. Nerve biopsy can help to exclude or confirm important differential diagnoses such as vasculitis and amyloidosis with different treatment options. However, nerve biopsy also has some limitations, such as variability in sampling and interpretation, potential complications, and lack of clear evidence-based guidelines for its use [12]. The report of the joint task force of the European Academy of Neurology and Peripheral Nerve Society therefore suggests consideration of biopsy:

- “In cases where CIDP is suspected but cannot be confirmed”
- “In cases where CIDP is suspected, but there is little or no response to treatment” [7].

The aim of this study was to investigate the indication for a nerve biopsy and its diagnostic and prognostic value in CIDP. We addressed the following research questions:

(Q1) What are the factors that influence the decision to perform a nerve biopsy in patients with suspected CIDP and what were the consequences of the biopsy on therapy?

(Q2) Can the outcome of the biopsy be predicted by clinical parameters to avoid unnecessary biopsies?

(Q3) Does the histological pattern of damage correspond to the electrophysiological pattern, and are electrophysiologically burnt-out nerves still histologically valuable?

(Q4) Can nerve biopsy predict the disease course and outcome in CIDP, and does the histological severity of damage correlate with the ODSS?

## Methods

We conducted a retrospective analysis of patients who underwent sural nerve biopsy at University Hospital—St. Josef-Hospital in Bochum, Germany, between 2012 and 2021, and who had a suspected diagnosis of CIDP and other immune-neuropathies. We also used data from the prospective Immunemediated Neuropathies Biomaterial and Data (INHIBIT) registry to compare patients with and without biopsy.

### Patient cohorts

A total of 190 sural nerve biopsies were analyzed. The first cohort (biopsy cohort) consisted of 163 patients who underwent a sural nerve biopsy for suspected immune neuropathies between June 2012 and January 2021. The indication for biopsy was based on the clinical judgment of the treating neurologist, who considered the patient’s history, symptoms, physical examination, laboratory tests, electrophysiology, cerebrospinal fluid (CSF) analysis, and response to immunotherapy.

The second cohort was recruited from the INHIBIT registry, established in 2019 in our institution, and consisted of 147 patients recruited until January 2021. Of these, 72 patients had a biopsy and 75 did not. The biopsy and INHIBIT cohort had an intersection of 45 patients. We assessed both cohorts for similarity to avoid a selection bias when comparing a non-biopsy and a biopsy group.

### Clinical data collection

We collected the following data from the biopsy cohort: age, sex, duration of symptoms at biopsy, and diagnosis categories: CIDP, other immune neuropathy, or no immune neuropathy. The CIDP category included typical CIDP and CIDP variants such as DADS, MADSAM, focal CIDP, pure motor CIDP, and pure sensory CIDP. The ‘other immune neuropathy’ category included paraneoplastic neuropathy, vasculitic neuropathy, and sarcoidosis. The ´no immune neuropathy´ category included hereditary neuropathy, diabetic neuropathy, toxic neuropathy, and other non-inflammatory causes. In addition, we collected data from nerve conduction studies (NCS) of sural nerves, tibial nerves, fibular nerves, radial nerves, ulnar nerves, median nerves regarding sensory nerve action potentials (SNAP), compound muscle action potentials (CMAP), nerve conduction velocity (NCV), F-wave-latencies, F-wave persistence, motor conduction block, abnormal temporal dispersion, motor distal latency prolongation, and distal CMAP duration. As the clinicians decided to perform the nerve biopsy based on the previous CIDP diagnostic criteria (electrophysiological EFNS/PNS criteria) [5] we evaluated the fulfillment of these criteria at the time of diagnosis.

We analyzed CSF protein level, pathological spontaneous activity (pSA) in electromyography, immunotherapy at biopsy and 6 months after biopsy (corticosteroids, intravenous immunoglobulins, plasma exchange, second line and escalation therapies such as azathioprine, mycophenolate-mofetil, ciclosporin or rituximab), response to immunotherapy at biopsy and 6 months after, and overall disability sum score (ODSS) at biopsy and 1 year after. The same data was collected for the INHIBIT cohort.

Treatment response was defined based on the change of treatment. For this purpose, the immunotherapy received prior to the biopsy was recorded until the day of the biopsy. No change in type, dose, or frequency of immunotherapy during this period was considered a positive treatment response, while a change in treatment was considered a negative treatment response. Patients who had not received immunotherapy until the biopsy were not included in this evaluation.

### Sural nerve biopsy and histological data collection

Sural nerve biopsy was performed under local anesthesia by a trained surgeon using a standard technique. Pathological assessment was carried out in the Institute of Neuropathology of the University of Duisburg-Essen, Germany, according to a standardized protocol. Specimens of each individual patient were divided into two parts. One part was fixed in 2.5% glutaraldehyde, embedded in epoxy resin, cut into semi-thin sections (0.5 µm) and stained with toluidine blue. The other half of the specimen was fixed in 10% formalin and embedded in paraffin. Subsequently, sections (3 µm) were cut and stained with hematoxylin and eosin (HE), Elastica-van-Gieson (EvG), and Congo red. To analyze the presence of immune cells, antibodies against CD3 (ABCAM), CD20 (Ventana Roche), CD45 (DAKO), and CD68 (DAKO) were used for immunohistochemical staining. All sections were examined by two neuropathologists independently using a Nikon microscope (Eclipse 80i). Biopsies were evaluated according to the following protocol:

- Acute axonal damage: occurrence of myelin ovoids
- Chronic axonal damage: clusters of axonal regeneration
- Acute demyelinating damage: signs of myelin degradation, such as striped myelin from the axon
- Chronic demyelinating damage: onion bulb formations, thin myelin layers
- Progressive neuropathy: axonal damage with signs of acute inflammatory damage
- Highly degenerated ‘burnt-out’ nerves: > 90% degradation of fibrils, almost only connective tissue
- Histopathological CIDP criteria
  - Signs of de- and remyelination
  - Axonal damage
  - Heterogeneous, ‘patchy’ damage of nerve fibrils
  - Endoneurial or epineurial lymphocytes (CD3, CD4, CD8, CD45, CD68)
  - Endoneurial (perivascular) macrophages clustering
- Histological outcome parameters:
  - diagnostic biopsy/unspecific biopsy
  - CIDP typical and atypical biopsy
  - highly degenerated “burnt-out” nerves / “not burnt-out” nerves

**Fig. 1:**
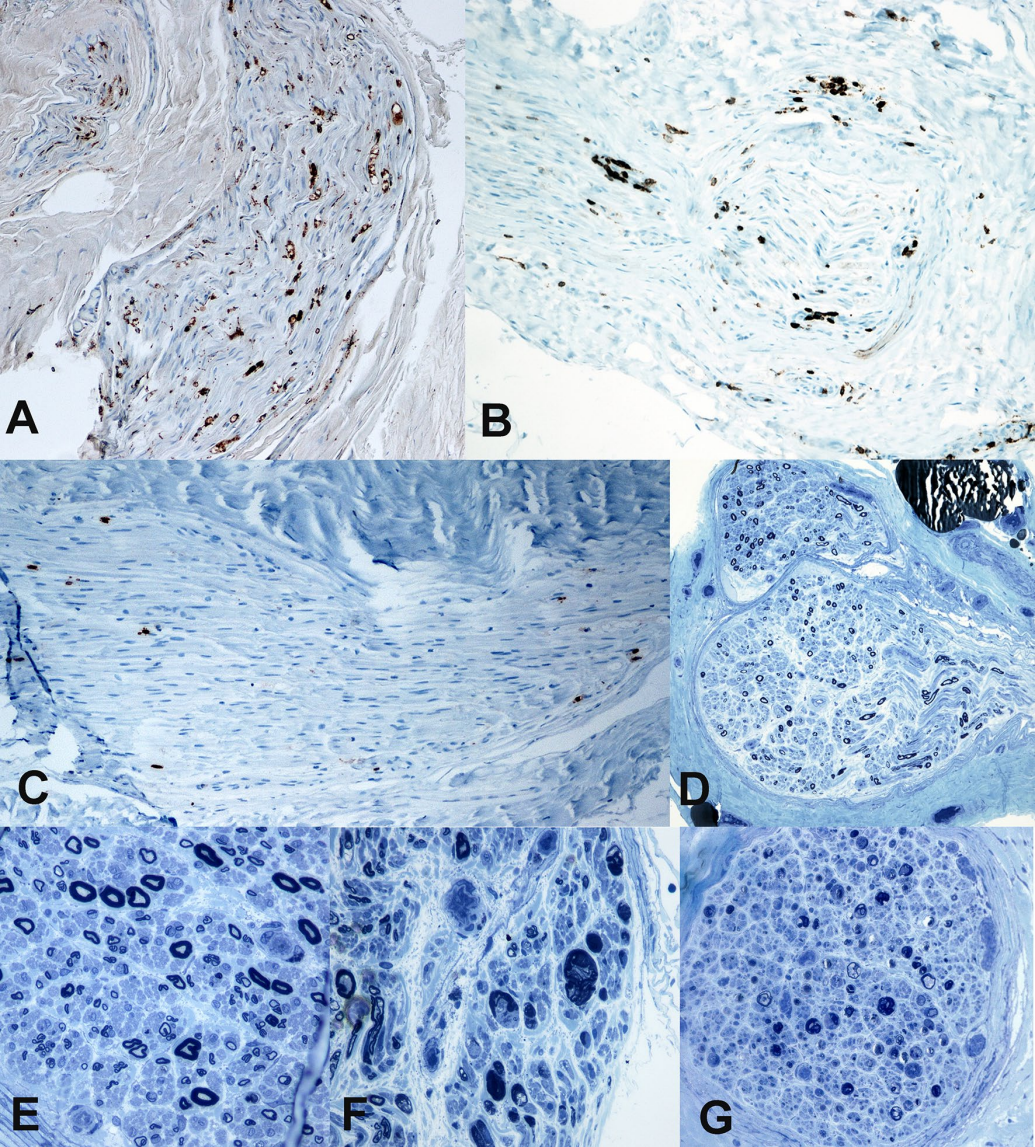
Representative histology findings. Representative histology findings. Immunoreaction against CD68 (**A**) shows an increased number of activated macrophages in endoneurium. Immunohistochemistry staining for CD45 (**B**) and CD8 (**C**) demonstrate infiltrating lymphocytes, in part consisting of cytotoxic T-cells. Toluidine blue stained semithin sections **D–G** demonstrate variation in fiber density, patchy distribution of axonal loss, endoneurial edema, attenuation and disruption of myelin sheaths, thin myelin layers as well as clusters of axonal regeneration and denuded axons, indicating chronic demyelinating and axonal damage. 100× magnification in **A**, **B**, **C**, **D**, and **G**. 200× magnification in **E** and **F**

Findings of nerve biopsies were available in written form after external implementation and were converted into evaluable variables (Table 1). Thereby, findings (demyelination, inflammation, onion bulb formation, axonal loss), severity grading (mild, moderate, severe), and regeneration and progression markers (florid or chronic pattern, remyelination or axonal regeneration) were evaluated. The damage pattern was classified as follows: ‘axonal loss’, ‘axonal damage’, ‘axonal damage with demyelinating signs’, ‘mixed axonal and demyelinating damage’, ‘demyelinating damage with axonal signs’, and ‘demyelinating damage’. Representative histological findings are shown in Fig. 1.

**Table 1:** List of histological variables and their evaluation method

Variable	Evaluation
Markers of progression	0 = no; 1 = yes
Regenerationtendency	0 = no; 1 = yes
Occurance of lymphocytes	0 = no; 1 = yes
Signs of microangiopathy	0 = no; 1 = yes
Diseased course	1 = acute; 2 = chronic
Histological diagnosis	0 = unspecific; 1 = CIDP; 2 = other than CIDP
Diagnostic biopsy	0 = unspecific; 1: yes; diagnostic value
Degree of damage	1 = mild; 2 = moderate, 3 = severe
Quantity of macrophages	1 = mild; 2 = moderate, 3 = severe
Mapping of neuronal damage pattern	1 = axon failure
	2 = mostly axonal
	3 = axonal + demyelinating
	4 = mixed
	5 = demyelinating + axonal
	6 = mostly demyelinating
Simplified mapping of neuronal damage patterns	1 = axon failure
	2 = axonal (+ demyelinating)
	3 = mixed
	4 = demyelinating (+ axonal)

### Statistics

Statistical analysis was performed using Excel (version 16.65, Microsoft, Redmond, USA) and IBM® SPSS Statistics (Armonk, NY, USA version 28.0.0.0). Data showed no normal distribution in the Shapiro–Wilk Test. Mann–Whitney U test and Kruskal–Wallis test were performed for analysis of metric and ordinally scaled variables. Nominal variables were tested using the Pearson chi-square test. We considered *p*-values < 0.05 as statistically significant (*p < 0.05; **p < 0.01; ***p < 0.001).

### Ethical approval

All procedures were in accordance with the ethical standard of the institutional research committee and with the 1964 Helsinki Declaration and its later amendments. The study was approved by the local ethics committee (vote no. 18-6534-BR, Ruhr-University Bochum, Germany) and was registered in the German Clinical Trials Register (Deutsches Register Klinischer Studien (DRKS), register name: Immune-mediated Neuropathies Biobank INHIBIT; register number: DRKS00024494).

## Results

### INHIBIT cohort allows representative comparison between biopsy and non-biopsy group

Characterization of the biopsy cohort (BSY) and the INHIBIT cohort (INH) showed similarity regarding clinical data (*p* > 0.05) such as protein levels in CSF (BSY: 632 ± 400 mg/dl; INH: 721 ± 639 mg/dl), sural SNAP (BSY: 1.6 ± 2.3 µV; INH: 2.6 ± 3.5 µV), ODSS at onset (BSY: 2.6 ± 1.9; INH: 2.5 ± 2.0), pSA (BSY: 59.7%; INH: 55.6%), and fulfilling of EFNS/PNS criteria (BSY: 76.3%; INH: 81.6%). INH was significantly younger at time of first manifestation (BSY: 58 ± 14; INH: 51 ± 13; *p* < 0.001), diagnosis (BSY: 61 ± 13; INH: 54 ± 13; *p* < 0.001) and biopsy (BSY: 61 ± 13; INH: 54 ± 13; *p* < 0.001). See Table 2.

Q1: Negative treatment response triggers the decision to perform a biopsy and positive findings trigger treatment escalation.

**Table 2:** Sociodemographic, epidemiological and clinical data of biopsy and INHIBIT cohort

	A: Biopsy cohort					B: INHIBIT cohort				A vs. B
	All	No immune-neuropathy	Other immune-neuropathy	CIDP	*p*-Values	All	Biopsy	No biopsy	*p*-Values	*p*-Values
*n* (%)	163 (100)	36 (22.1)	24 (14.7)	103 (63.2)	–	147 (100)	72 (49.0)	75 (51.0)	–	–
Age at disease onset, years *M* ± SD	57.8 ± 13.5	55.7 ± 16.1	54.3 ± 16.7	59.3 ± 11.5	0.218	50.8 ± 12.6	51.0 ± 11.9	50,6 ± 13.2	0.594	< 0.001***
Age at diagnosis, years *M* ± SD	61.2 ± 13.4	58.8 ± 16.1	57.0 ± 16.0	62.9 ± 11.3	0.099	53.8 ± 12.8	54.2 ± 11.6	53.4 ± 14.0	0.773	< 0.001***
Age at biopsy, years *M* ± SD	61.4 ± 13.4	58.9 ± 16.1	57.5 ± 16.2	63.2 ± 11.3	0.117	–	53.9 ± 12.6	–	–	< 0.001***
Sex, *n* male/female	116/47	26/10	15/9	75/28	0.596	113/34	58/14	55/20	0.343	> 0.05
Disease duration at biopsy, month *M* ± SD	43.5 ± 47.6	38.0 ± 41.1	37.8 ± 61.9	46.7 ± 46.1	0.053	–	41.5 ± 46.3	–	–	> 0.05
Cerebrospinal fluid protein levels, mg/l, *M* ± SD – Biopsy cohort: *n* = 147 – INHIBIT cohort: *n* = 112	632 ± 400	557 ± 246	667 ± 513	651 ± 416	0.773	721 ± 639	680 ± 596	766 ± 686	0.067	> 0.05
SNAP N. suralis, µV, Mean ± SD and median (IQR) – Biopsy cohort: *n* = 151 – INHIBIT cohort: *n* = 106	1.6 ± 2.3 0 (2.6)	1.4 ± 2.0 0 (2.8)	2.5 ± 3.5 0 (4.8)	1.5 ± 1.9 0.785 (2.4)	0.783	2.6 ± 3.5 1.45 (3.89)	2.2 ± 2.8 1.31 (3.11)	2.9 ± 3.9 1.53 (4.58)	0.715	> 0.05
Pathological spontaneous activity, *n* (% sub-cohort) – Biopsy cohort: *n* = 154 – INHIBIT cohort: *n* = 133	92 (59.7)	18 (11.6)	15 (9.7)	59 (38.3)	0.783	74 (55.6)	39 (59.1)	35 (52.2)	0.472	> 0.05
ODSS at disease onset (*M* ± SD)	2.6 ± 1.9	2.7 ± 2.2	3.0 ± 2.8	2.4 ± 1.6	0.88	2.5 ± 2.0	2.7 ± 2.1	2.4 ± 1.9	0.447	> 0.05
ODSS at biopsy (*n* = 24, *M* ± SD)	2.6 ± 1.8	2.6 ± 2.2	3.0 ± 2.2	2.5 ± 1.5	0.51	–	2.8 ± 1.8	–	–	> 0.05
Fulfilment of EFNS/PNS criteria at biopsy, *n* (% sub-cohort) – Biopsy cohort: *n* = 160 – INHIBIT cohort: *n* = 114	122 (76.3)	26 (76.5)	11 (47.8)	85 (82.5)	0.002**	93 (81.6)	47 (85.5)	46 (78.0)	0.206	> 0.05
Fulfilment EFNS/PNS criteria at disease onset (*n* = 89), *n* (% sub-cohort)	–	–	–	–	–	68 (76.4)	33 (76.7)	35 (76.1)	0.987	–
Diabetes (*n* = 140)? *n* (% sub-cohort)	–	–	–	–	–	16 (10.9)	8 (11.6)	8 (11.3)	0.725	–
HbA1c > 6.5% (*n* = 44)? *n* (% sub-cohort)	–	–	–	–	–	4 (9.1)	1 (6.3)	3 (10.7)	0.858	–

**Fig. 2:**
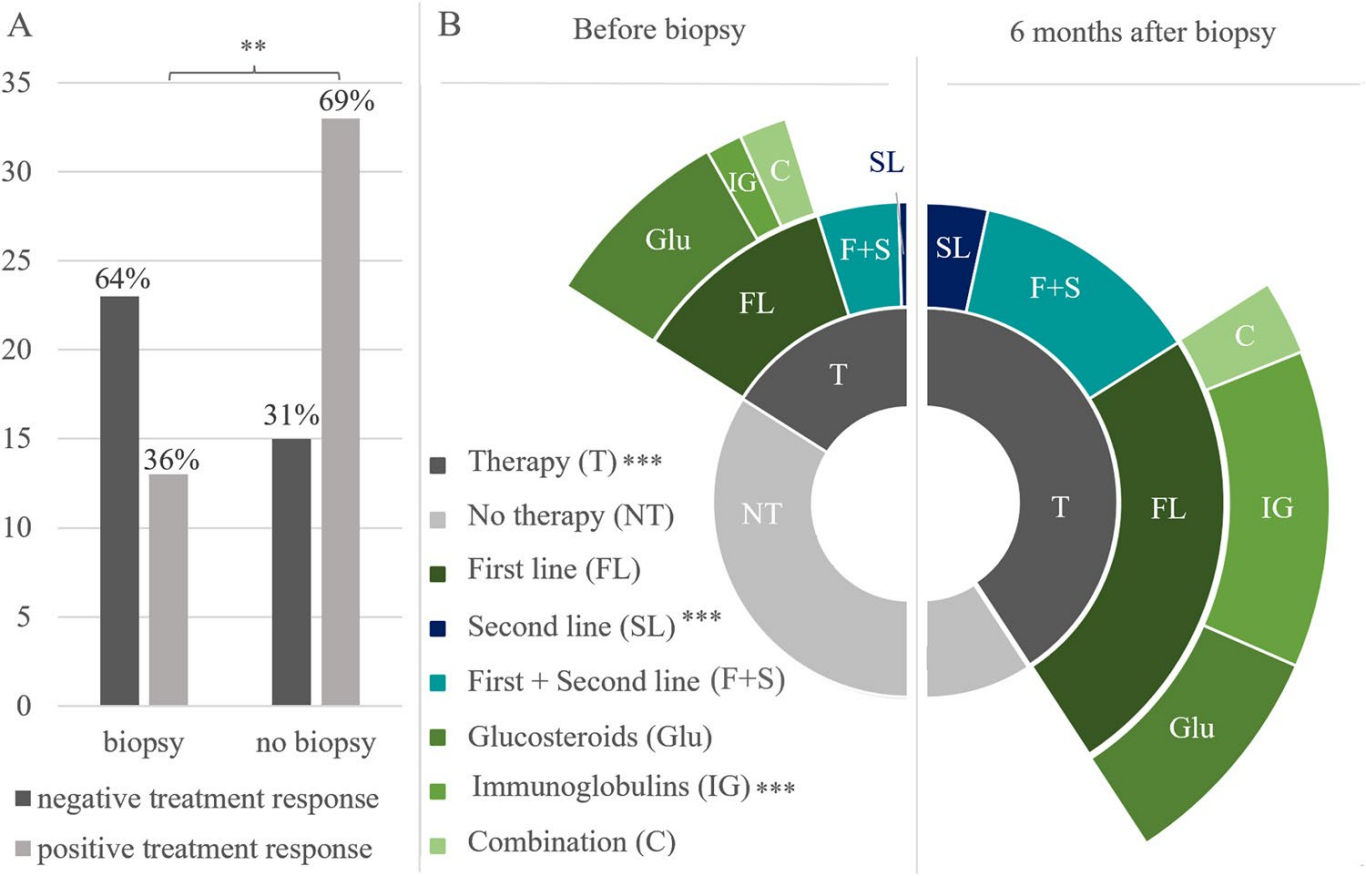
Data of treatment. **A** Treatment response of patients with and without nerve biopsy. No change in type, dose, or frequency of immunotherapy was considered a positive treatment response, while a change in treatment was considered as negative treatment response. Significant difference in positive treatment response between patients with and without biopsy (*p* = 0.003). **B** Distribution of therapeutics before and 6 month after biopsy. *N* = 103 patients. Before biopsy and after: *T* = 33/85 (*p* < 0.001); NT = 68/17; F + S = 9/26; SL = 10/33 (*p* = 0.0001); FL = 23/51; Glu = 16/19; IG = 3/23 (*p* = 0.0001); C = 4/6. Treatment with intravenous Immunoglobulins and second-line treatments were significantly increased after biopsy

Treatment response was compared between patients with and without biopsy in the INHIBIT cohort. Biopsied patients were significantly less likely to respond to immunotherapies (*n* = 13, 36%) than non-biopsied patients (*n* = 33, 69%; *p* = 0.003, Fig. 2A). Six months after biopsy, significantly more patients received immunotherapy than before (34% vs. 84%, *p* < 0.001). The ratio of glucocorticoids to immunoglobulins shifted significantly in favor of immunoglobulins (before: 24.3% glucocorticoids, 3.9% immunoglobulins; after: 38.8% glucocorticoids, 49.5% immunoglobulins, *p* = 0.0001). The proportion of second-line therapies increased (before: *n* = 10, after: *n* = 33; +22.3%, *p* = 0.0001) (Fig. 2B).

Q2: Outcome of the biopsy cannot be predicted by clinical parameters to avoid unnecessary biopsies.

We investigated in the biopsy cohort whether clinical parameters could predict results of nerve biopsy. We analyzed disease duration, CSF protein, sural SNAP, pSA, and ODSS and compared them with the aforementioned biopsy outcome parameters.

**Fig. 3:**
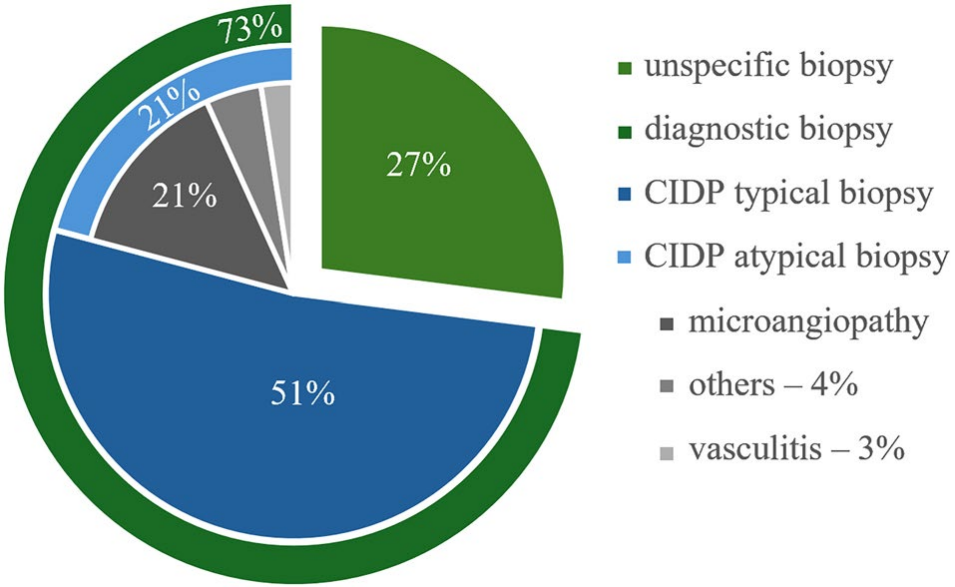
Diagnosis within the biopsy cohort (*n* = 163 patients). 119 biopsies were diagnostically valuable (73%). Of these, 85 (51.1%) biopsies showed histologically a CIDP and 34 (20.9%) showed other diagnosis such as vasculitis (*n* = 4, 2.5%), microangiopathy (*n* = 23, 14.1%), and others (*n* = 7, 4.3%)

119 (73%) biopsies showed a specific diagnosis and 44 (27%) were unspecific. 85 (51%) showed a CIDP and 34 (21%) showed other diagnoses such as vasculitis (*n* = 4, 3%), microangiopathy (*n* = 23, 14%), and others (*n* = 7, 4%). Of all biopsies, 15 (9%) showed ´burnt-out´ nerves while 148 (91%) did not (Fig. 3).

There were no significant differences in clinical variables between diagnostic and unspecific biopsies. Patients had similar disease duration, CSF protein, sural SNAP, pSA occurrence, and ODSS (Table 3). Comparisons of clinical variables with the outcome parameters histologically CIDP (h-CIDP) and histologically non-CIDP (hn-CIDP) showed no significance (Table 3). Regarding histologically burnt-out and non-burnt-out nerves, a different picture emerges. There were no significant differences in terms of disease duration (*burnt-out*: 58.2 ± 65.5 months; *not burnt-out:* 42.0 ± 45.4 months; *n* > 0.05) and pSA occurrence (*burnt-out:* 11/12%; *not burnt-out:* 81/88%; *n* > 0.05), but CSF protein (*burnt-out*: 438 ± 199 mg/dl; *not burnt-out*: 651 ± 410 mg/dl; *p* = 0.04), sural nerve SNAP (*burnt-out*: 0 ± 0 µV; *not burnt-out*: 1.8 ± 2.3 µV; *p* < 0.001) and ODSS (*burnt-out*: 3.4 ± 1.6; *not burnt-out*: 2.5 ± 1.8; *p* = 0.025) were significantly different (see Table 3).

Q3: Patients with mostly demyelinating histological damage more often fulfill FNS/PNS criteria and suralis loss should not be disqualified for nerve biopsy.

**Table 3:** Comparison of histological sub-groups

	Diagnostic	Unspecific	*p*-Values	Histologically CIDP	Histologically non CIDP	*p*-Values	Burnt-out	Not burnt-out	*p*-Values
*n* (%)	119 (73)	44 (27)	–	85 (51)	34 (21)	–	16 (9)	148 (91)	–
Disease duration at biopsy, month *M* ± SD	43 ± 48	45 ± 47	> 0.05	44 ± 49	40 ± 47	> 0.05	58.2 ± 65.5	42.0 ± 45.4	> 0.05
Cerebrospinal fluid protein levels, mg/l, *M* ± SD	669 ± 429	534 ± 292	> 0.05	677 ± 471	629 ± 314	> 0.05	438 ± 199	651 ± 410	0.04 *
Sural SNAP, µV, *M* ± SD, (IQR)	1.5 ± 2.1 0 (2.6)	2.0 ± 2.6 0 (2.7)	> 0.05	1.4 ± 1.9 0 (2.5)	1.8 ± 2.6 0 (2.8)	> 0.05	0 ± 0 0 (0)	1.8 ± 2.3 1 (2.8)	< 0.001 ***
Occurrence of pathological spontaneous activity, *n* (%)	64 (70)	28 (30)	> 0.05	45 (70)	19 (30)	> 0.05	11 (12%)	81 (88%)	> 0.05
ODSS, *M* ± SD	2.5 ± 1.6	3.0 ± 2.8	> 0.05	2.5 ± 1.6	2.4 ± 1.5	> 0.05	3.4 ± 1.6	2.5 ± 1.8	0.025 *

Significant differences were found between histologically burnt-out and not-burnt-out nerves in CSF protein levels (p = 0.04), sural SNAP (*p* < 0.001), and ODSS (*p* = 0.025)

**Figure 4:**
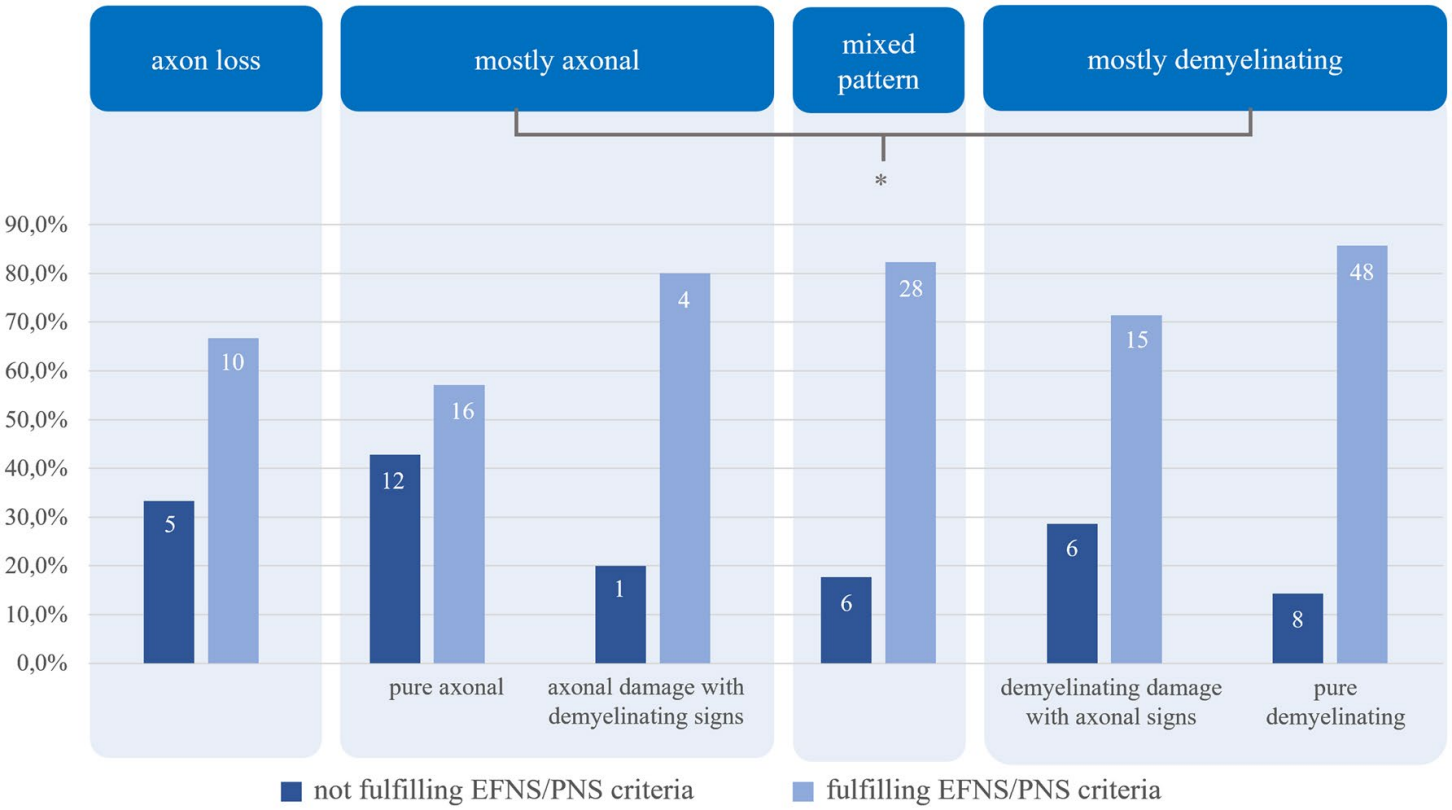
EEFNS/PNS fulfillment in the biopsy cohort and distribution of histological damage pattern. Comparisons show a significant higher fulfillment of criteria in patients with mostly demyelinating damage pattern (*p* = 0.027)

We analyzed 159 electrophysiologically examined patients for the fulfillment of the FNS/PNS criteria. In total, 121 (76%) met the criteria and 38 (24%) do not. When comparing the histological groups ‘predominantly axonal damage’ with ‘predominantly demyelinating damage’, there was a significantly higher fulfillment of the EFNS/PNS criteria in the histologically predominantly demyelinating damage pattern (*p* = 0.027). Detailed information is displayed in Fig. 4. After filtering all biopsies for a CIDP diagnosis, it was found that CIDP-typical histology findings fulfilled the EFNS/PNS criteria in 77% of cases and did not in 23%.

**Fig. 5:**
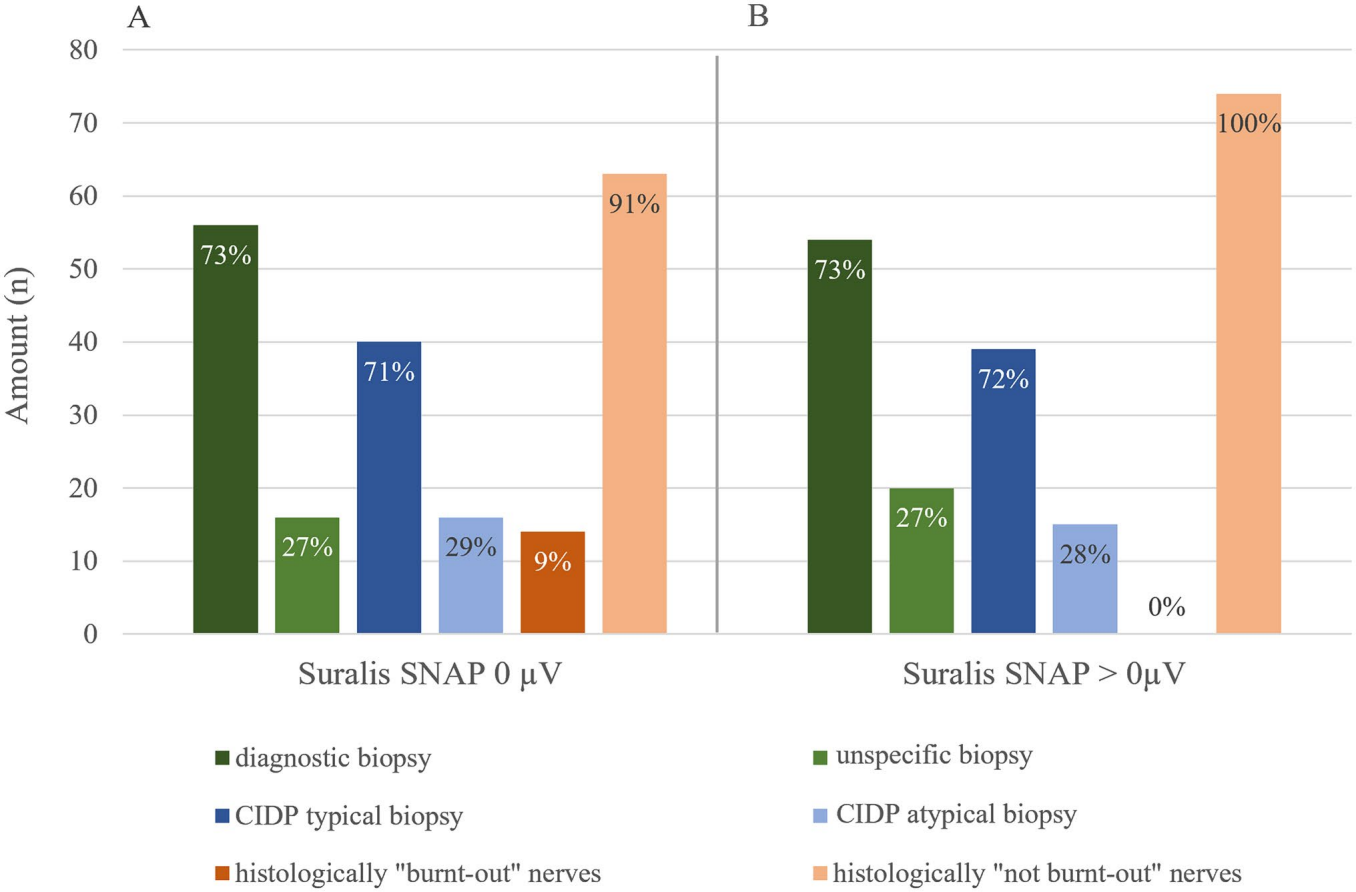
Comparison of histological outcome parameters depending on the electrophysiological measurement result of the N. suralis of 151 patients. **A** Distribution of histological outcomes in patients with electrophysiological suralis SNAP of 0 µV (*n* = 77). **B** Distribution of histological outcomes in patients with electrophysiological suralis SNAP > 0 µV (*n* = 74). Corresponding colors matching histological outcome parameters. Green = diagnostic or unspecific histology. Blue = histologically CIDP or CIDP atypical biopsy. Orange = histological burn-out nerves or not burn-out nerves. The percentages always refer to the group. There were no significant differences in histological outcome parameters. SNAP of 0 µV showed only 9% histologically burn-out pattern

Besides, a total of 151 patients of the biopsy cohort had documented NCS. Of these, 77 (51%) show a sural SNAP of 0 µV and 74 (49%) greater than 0 µV. For a sural SNAP of higher than 0 µV, a biopsy was able to provide a diagnosis in 54 (73%) patients and no diagnosis in 20 (27%). 39 patients (72%) received the histological diagnosis of CIDP and 15 (28%) other diagnosis. None of the nerves with SNAP greater 0 µV showed a histologically burnt-out pattern. In addition, biopsies were able to provide a histological diagnosis in 56 (73%) of cases with a sural SNAP of 0 µV and no diagnosis in 21 (27%). Of these, 40 patients (71%) received the histological diagnosis of CIDP and 16 (29%) another diagnosis. Only 14 (9%) of the biopsies with sural SNAP of 0 µV were histologically burnt-out (63, 91%). In general, 79 of 151 (52%) electrophysiologically examined patients received the histological diagnosis CIDP (Fig. 5).

Q4: Histological damage does not correlate with clinical severity but progression markers enable a prognosis of physical impairment.

**Fig. 6:**
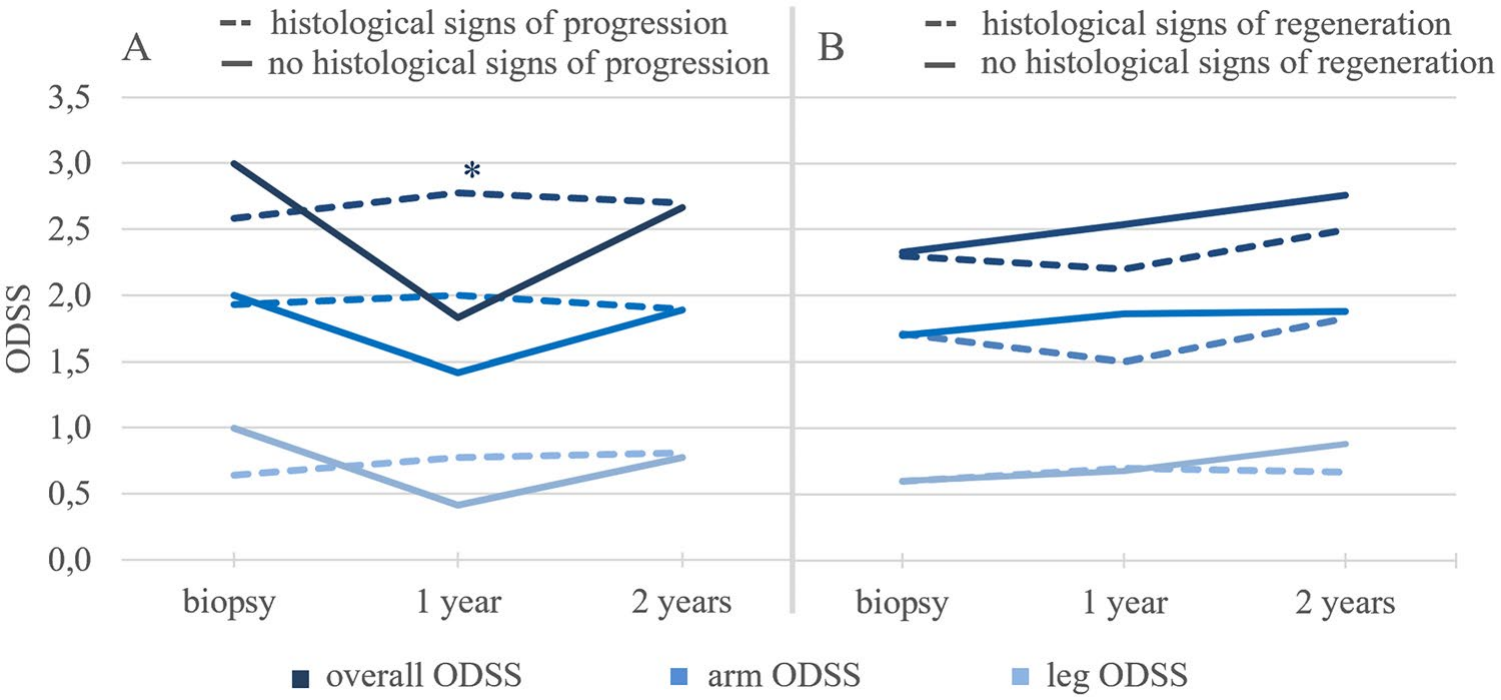
Comparison of ODSS (overall, arm, leg) of patients with and without histological signs of progression. (**A**) and regeneration (**B**). **A** A total of 92 datasets was examined for histological signs of progression at time of biopsy, 68 after a year, and 45 after 2 years. After 1 year, patients with signs of progression in histology had significantly higher overall ODSS (with progression markers: 2.77 ± 2.05; without progression markers 1.83 ± 1.57; *p* = 0.028). **B** A total of 76 datasets was examined for histological signs of regeneration at time of biopsy, 60 after a year, and 40 after 2 years

We compared the severity of histological damage with the clinical impairment. Of 153 biopsies, 39 (26%) showed mild, 34 (22%) moderate, and 80 (52%) severe damage. The following numbers are displayed as mean ± SD. Mild damage: arm ODSS = 0.8 ± 0.9; leg ODSS = 2.0 ± 1.6; total ODSS = 2.7 ± 2.2. Moderate damage: arm ODSS = 0.6 ± 0.7; leg ODSS = 2.0 ± 1.6; total ODSS = 2.5 ± 1.6. Severe damage: arm ODSS = 0.7 ± 0.9, leg ODSS = 1.8 ± 1.3; total ODSS = 2.6 ± 1.7. There were no significant differences in ODSS values for the different severity grades (*n* > 0.05). Besides, the prognostic significance of nerve biopsies was determined based on the ODSS of CIDP one and 2 years after biopsy. A total of 76 datasets were analyzed for signs of regeneration at the time of biopsy. Due to patients dropping out during the observation period, 60 datasets remained at 1 year and 40 at 2 years. Comparison of patients with and without histologic evidence of regeneration showed no significant differences in the level of overall, arm, or leg ODSS. There were significant differences in ODSS between patients with and without histologic evidence of progression. 92 datasets were analyzed for histologic signs of progression at the time of biopsy, 68 datasets remained at 1 year, and 45 at 2 years. It is shown that patients with histological progression markers had significantly higher ODSS at 1 year (with signs of progression: ODSS = 2.8 ± 2.1; without signs of progression: ODSS = 1.8 ± 1.6; *p* = 0.028). While histological progression markers allowed a prognosis on physical impairment, histological regeneration markers did not (Fig. 6).

## Discussion

The aim of this study was to investigate the indications and benefits of sural nerve biopsy in a cohort of patients with CIDP and other suspected immune-mediated neuropathies. Regarding the factors that led to the performance of nerve biopsy, our data show that sociodemographic or clinical parameters did not play a role. With a delay of more than 40 months after disease onset, the biopsy also had no significance in the initial diagnosis of CIDP but had a confirmatory role. It was mainly the previous lack of response to therapy that led clinicians to decide to perform a nerve biopsy. According to the data, the biopsied patient cohort appeared to have a lower treatment response prior to their biopsy compared to a non-biopsy patient cohort (36% vs. 69%; *p* = 0.003).

In addition, it was observed that the proportion of patients receiving therapy increased significantly after a CIDP-typical biopsy (34% vs. 83.5%; *p* < 0.001). It was also observed that the choice of the drug after biopsy was significantly more often immunoglobulins, changing from corticosteroids. Furthermore, the number of second-line immunotherapies also increased significantly (before biopsy *n* = 10, after biopsy *n* = 33; +22.3%, *p* = 0.0001). Biopsy evidence of higher-grade nerve damage with immune cell infiltration may have convinced treating physicians that the disease was a severe immune neuropathy. As a result, patients were treated more frequently and aggressively after biopsy.

It is of note, that 27% of the biopsies were unspecific. Here, we evaluated signs of de- and remyelination, axonal damage, heterogeneous, ‘patchy’ damage of nerve fibers, endoneurial or epineurial lymphocytes and endoneurial (perivascular) macrophages. Other parameters, some of which are derived from or related to the above-mentioned ones, include endoneurial macrophage clustering, macrophage-mediated demyelination, increased endoneurial DC8-positive cytotoxic T cells, and endoneurial edema were not addressed. Furthermore, the EAN/PNS Task Force recommends in its 2021 criteria the analysis of thinly myelinated or demyelinated internodes in teased fibers, which was not conducted in this study.

In this study, based on histological terms the quality and quantity of nerve fibers were not the reason for the high amount of unspecific biopsies. Rather, the biopsy could provide indications for the presence of a CIDP, but the criteria for the diagnosis of a CIDP were not fulfilled. Nevertheless, it is important to mention that the significance of a biopsy always depends on its quality. In particular, the number of nerve fascicles, the size of the specimen, and the occurrence of artifacts such as blood or bruises play a crucial role. Other studies have come to similar conclusions and therefore recommend careful selection of patients since nerve biopsies also have complications [13, 14].

Furthermore, our study showed that the diagnostic sensitivity of nerve biopsy cannot be predicted by clinical or laboratory parameters. ODSS, NCS, CSF protein, and pSA were not able to predict a high diagnostic sensitivity of nerve biopsy. However, Dyck and Tracy pointed out that a negative biopsy does not rule out CIDP, and if CIDP is suspected despite a negative biopsy, an attempt at therapy should be considered [15].

Surprisingly, it has been shown that even sural nerve biopsy with non-elicitable SNAP may have diagnostic value. 73% of patients with a sural SNAP of 0 µV had a diagnostical biopsy. Despite a sural SNAP of 0 µV, the frequency of CIDP-typical findings on histology was similar (72%) to that of patients with a sural SNAP higher than 0 µV. In view of this result, patients with electrophysiologically exhausted nerves should be considered for nerve biopsy if they are refractory to treatment. Selection of the nerve for biopsy has been reduced by many sources to the recommendation to select a clinically and electrophysiologically affected nerve [5, 16–19]. However, our data show that even severely electrophysiologically affected nerves have histologic value and should be considered for biopsy.

These data are supported by analyses that fit the EFNS/PNS criteria within the biopsy cohort. In the case of histological axonal loss, the criteria were still met in 66.6% of cases. Furthermore, patients with histologically predominantly demyelinating damage were significantly more likely to meet the EFNS/PNS criteria than patients with a predominantly axonal damage pattern (*p* = 0.027). In addition, after filtering for histological CIDP diagnosis 23% of patients did not fulfill the EFNS/PNS criteria. Due to the characteristic histological presentation, therapy initiation or escalation we believe is warranted in these patients, even though they did not meet the ENFS/PNS criteria. It is important to mention here that the data were collected when these criteria were valid. Indeed, the current EAN/PNS guidelines might lead to a different conclusion, as they introduce more supportive criteria for diagnosing CIDP. While nerve biopsy may not be necessary in all cases, particularly when other supportive criteria are strongly met, the presence of CIDP-typical findings in a nerve biopsy can significantly strengthen the diagnostic certainty of CIDP, as emphasized in the current EAN/PNS guidelines. Our results support the sensitivity of nerve biopsy in the diagnosis of CIDP.

These findings may suggest that the electrophysiologic severity of nerve damage predicts the histologic severity. However, further analysis proves that this is not the case. We classified the histologic severity of damage into three levels and assigned the ODSS of each patient to these levels. No significant difference was found between the histologic severity in terms of ODSS. Thus, the clinical phenotype of the patients could not be inferred from the histological severity. Conversely, a clinically severely affected patient may have less severe histologic damage.

An analysis of the ODSS in relation to histological regeneration and progression markers (combination of axonal damage and inflammatory infiltration) showed that patients with progression markers had a significantly worse overall ODSS 1 year after biopsy than patients without progression markers. Thus, nerve biopsy may also have prognostic value for patients and their subsequent disease course. However, our data also showed that this correlation was no longer detectable in the second year. In addition, our data also showed that histological marker of regeneration did not predict ODSS. This could be due to the fact that the number of patients followed up decreased over the observation period. Longer follow-up periods may be required to reliably predict clinical prognosis using progression and regeneration markers.

The main limitation of these findings is that treatment response could only be determined retrospectively and indirectly through the number of therapy changes. Nevertheless, the results are relevant because they are the first in the literature to compare biopsied and non-biopsied patients. In addition, nerve biopsies in case of insufficient or lack of treatment response are also discussed in the current EAN/PNS criteria, as this could indicate another underlying diagnosis [7]. A number of studies have also shown that nerve biopsy has an impact on the use of therapies. Gabriel et al. described treatment decisions based on biopsy results [20]. Ruth et al. also evaluated the impact of nerve biopsy on treatment in a cohort of 67 patients [21]. Therefore, our results are in line with existing literature. Another limitation of this study is that not all histologically relevant examinations could be retrospectively analyzed. In particular, the absence of teased fiber analysis may have contributed to the high proportion of unspecific biopsies.

In conclusion, a sural nerve biopsy should be considered in case of treatment refractory suspected CIDP. It should be noted that the diagnostic sensitivity of nerve biopsy cannot be predicted by clinical and electrophysiological parameters. Valuable diagnostic information can be obtained from biopsy in 73% of cases with extinguished sural SNAP. Biopsies with signs of progression may provide prognostic information for the short-term disease course. Further studies with longer follow-up are needed to confirm this prognostic value.

## Data Availability

Data collected from this study are available by e-mailing rafael.klimas@rub.de.

## Declarations

### Patient and public involvement

The patients gave spoken and written consent for publication and participation in the study.
